# Supplementary material for: Effect of Rootstock Genotype and Arbuscular Mycorrhizal Fungal (AMF) Species on Early Colonization of Apple
Source: Plants (Basel). 2024 May 16;13(10):1388. doi: 10.3390/plants13101388 (PMC11125189; doi:10.3390/plants13101388)
Supplement: Supplementary file 1 [file plants-13-01388-s001.zip › plants-2942802-supplementary.pdf]

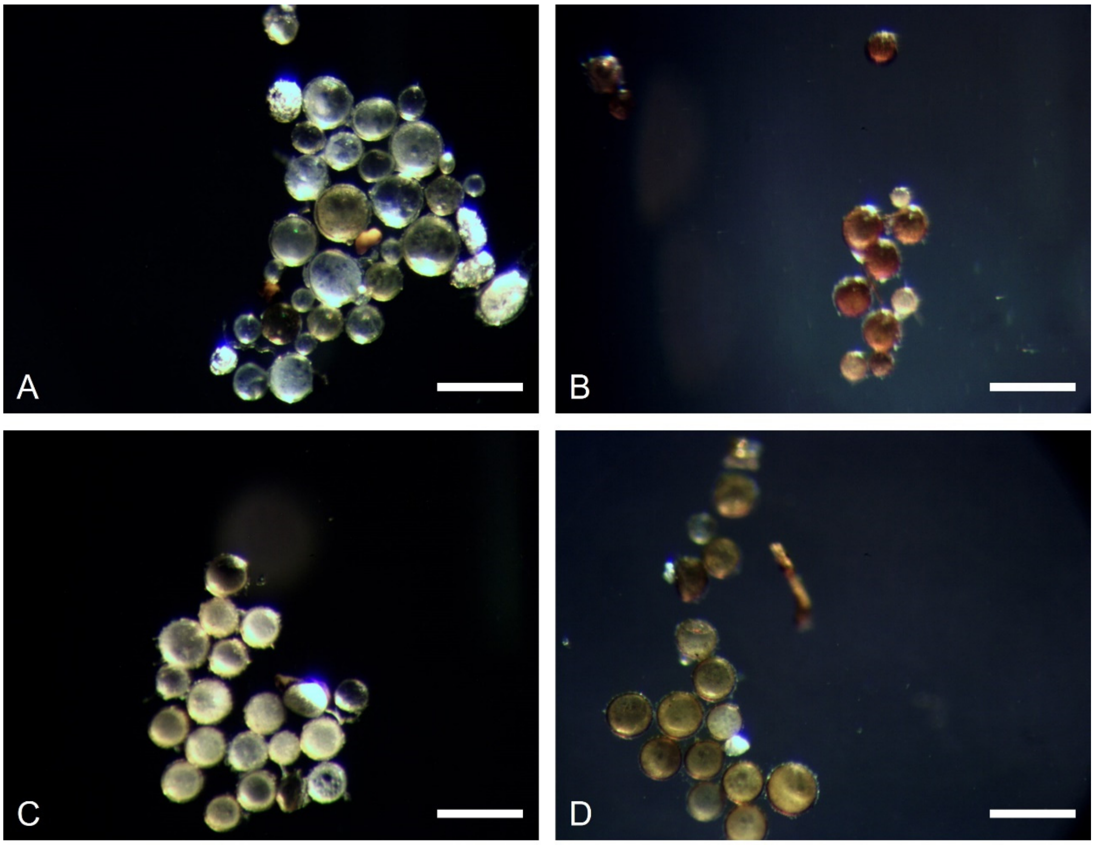

Figure S1. Spores of (A) *Rhizophagus irregularis*, (B) *Septoglomus deserticola*, (C) *Claroideoglomus claroideum* and (D) *Claroideoglomus etunicatum*; scale bars = 200 µm

>HG_003_ST1130121_E12

TAACATCGTCTGATTCCTAGTCGGCATAGTTTATGGTTAAGACTACGACGGTATCTGATCGTCTTCGAT
CCCCTAACTTTCGTTCTTGATTAATGAAAACATCCTTGGCAAATGCTTTCGCAGTAGTTAGTCTTCAAT
AAATCCAAGAATTTCACCTCTGACAATTGAATACTAATGCCCCCAACTATCCCTATTAATCATTACGGT
GATCCTAGAAACCAACAAAATAGAATCATGCCGTCCTATTTTATTATTCCATGCTAATGTATTCAAGCA
AAATGCCTGCTTTAAACACTCTAATTTTTTCAAAGTAAAGGTCCTGGTTCCCCGTGACACCCAATTAAG
GGCATCGCGGTTCTCCAGAAGGTGAGAAATTGACTACACCAGTTCATACCCCTTAGGGCACGACCGAT
GTGTCAATCCCGAAATTCAACTACGAGCTTTTTAACTGCAACAACTTTAATATACGCTATTGGAGCTGG
AATTACCGCGGCTGCTGGCACCAGACTTGCCCTCCAATTGTTCCTCGTTAAGGGATTTAAATTGTACTC
ATTCCAATTACGAGACCCGAAAGAGCCCCGTATTGTTATTTATTGTCACTACCTCCCCGTGTCGGGATT
GGGTAATTTGCGCGCCTGCTGCCTTCCTTGGATGTGGTAGCCGTTTCTCAGGCTCCCTCTCCGGAATCG
AACCCTAATTCCCCGTTACCCGTTACCACCATGGTAGGCCTCTATCCT

Accession	Description	Max score	Total score	Query coverage	E-value	Max identity
Y17639	Claroideoglomeraceae Claroideoglomus etunicatum VTX00193	734	734	100.0%	0	99.9%

Figure S2: 18S rRNA sequence data obtained from a single spore isolated from the commercial inoculum expected to contain *C. etunicatum*. The MaarjAM database (https://maarjam.ut.ee/?action=sBlast&id=132258) was used to confirm AMF species identity. Quality sequence data (with clear nucleotide peaks) could not be obtained for any other isolates.

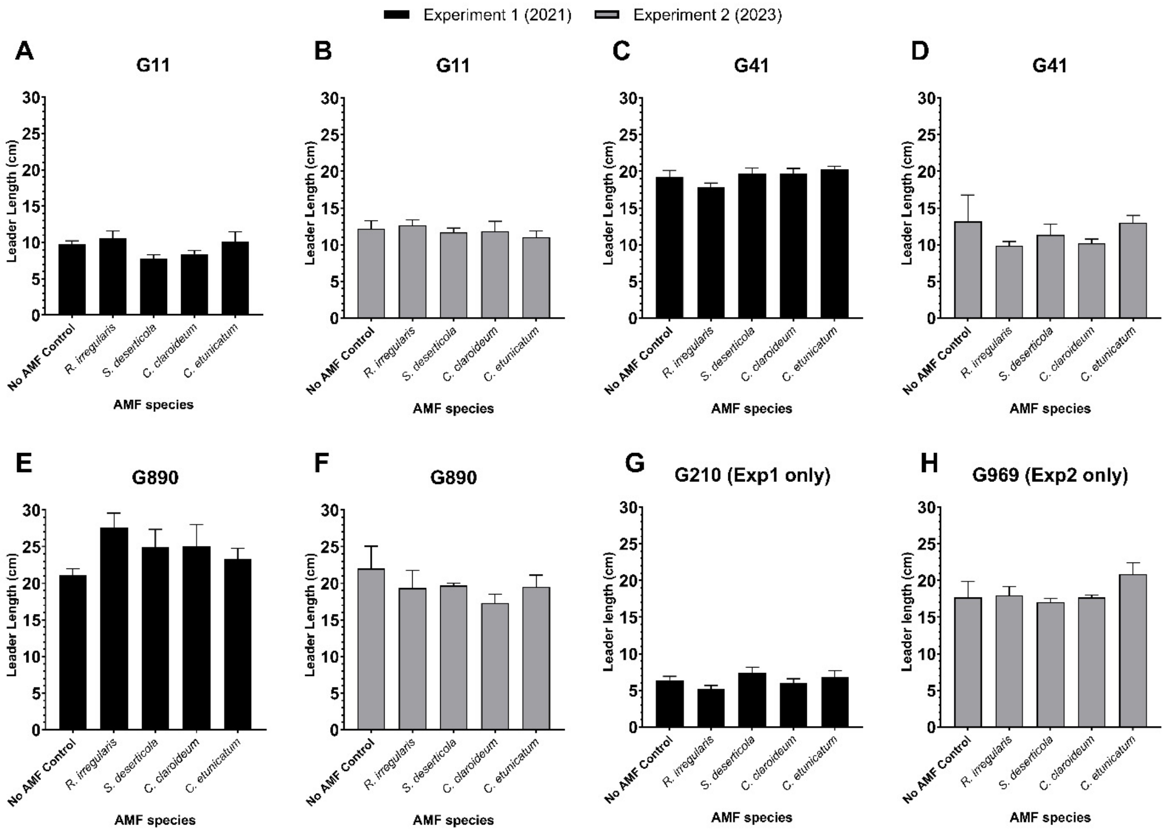

Figure S3: Leader shoot lengths of apple rootstock genotypes inoculated with different species of AMF, 5 weeks after inoculation in Exp 1 (black bars) and Exp 2 (grey bars). Different letters indicate statistically significant differences ($p < 0.05$) in leader length within each rootstock genotype (Two-way ANOVA on transformed (y=ln(y)) data followed by Tukey's multiple comparisons tests). Data represents the mean of 7 replicates (Exp 1) and 3 replicates (Exp 2). Error bars represent the standard error of the mean.

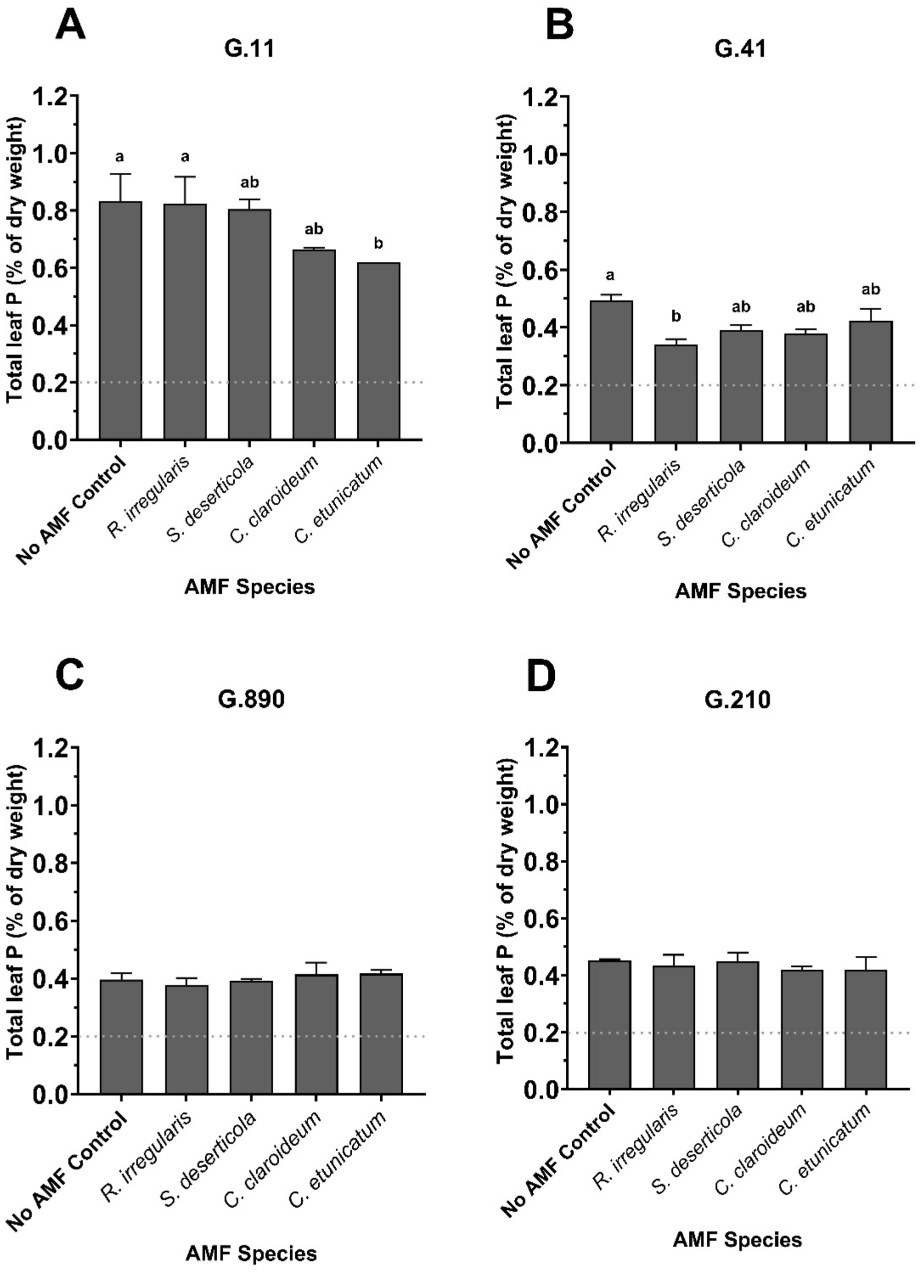

Figure S4: Total leaf foliar P (% of dry weight) in 4 different apple rootstocks inoculated with different species of AMF, 5 weeks after inoculation in Exp 1. Letters indicate statistical differences ($p<0.05$) in total leaf P (% of dry weight) observed according to two-way ANOVA followed by Tukey's multiple comparisons test. The dashed horizontal line indicates adequate P nutrition (> 0.2%). Data represents the mean of 3 samples pooled from 7 replicate plants.

Table S1: Mean percentage of AMF colonization for each apple rootstock genotype x AMF treatment combination at 5 weeks post-inoculation in Exp1 (2021) and at 2-, 5- and 8-weeks post-inoculation in Exp2 (2023). At each timepoint, data was compared across rootstocks for a given AMF species; rootstocks with the same letter were not significantly different (Dunnett's T3 tests following Welch's ANOVA test). Columns without letters indicate no significant differences. Exp1 values are based on 7 biological replicates per rootstock genotype/AMF treatment combination. Exp2 values are based on 3 biological replicates (x 3 technical replicates each).

Timepoint	Treatment ▶ / Genotype ▼	*R. irregularis*	*S. deserticola*	*C. claroideum*	*C. etunicatum*
Exp1 – 5 weeks	G.11	6.29	5	5 [ab]	4.29
Exp1 – 5 weeks	G.41	4.57	8.43	6.57 [ab]	5.71
Exp1 – 5 weeks	G.210	3.86	4	2.57 [a]	4.71
Exp1 – 5 weeks	G.890	4.43	5.14	7.71 [b]	4.29
Exp2 – 2 weeks	G.11	0.47 [a]	0	0.41	0.2 [a]
Exp2 – 2 weeks	G.41	4.87 [b]	0.17	1.4	1.98 [b]
Exp2 – 2 weeks	G.969	9.56 [ab]	0	0.11	9.6 [c]
Exp2 – 2 weeks	G.890	4.82 [ab]	1.13	0.77	6.88 [abc]
Exp2 – 5 weeks	G.11	10.16	1.07	25.28	40.73 [a]
Exp2 – 5 weeks	G.41	14.31	0.27	12.85	28.86 [ab]
Exp2 – 5 weeks	G.969	5.73	0	0	3.74 [b]
Exp2 – 5 weeks	G.890	9.27	0	5.9	7.13 [b]
Exp2 – 8 weeks	G.11	0 [a]	0 [a]	0.36 [a]	0 [a]
Exp2 – 8 weeks	G.969	29.09 [b]	4.59 [ab]	33.42 [b]	25.74 [b]
Exp2 – 8 weeks	G.890	4.15 [b]	9.32 [b]	36.66 [b]	42.85 [b]
